# Editorial: The cerebellar involvement in non cerebellar pathologies

**DOI:** 10.3389/fncel.2023.1232155

**Published:** 2023-06-13

**Authors:** Pellegrino Lippiello, Francesca Montarolo, Keiko Tanaka-Yamamoto, Eriola Hoxha

**Affiliations:** ^1^Department of Pharmacy, School of Medicine and Surgery, University of Naples Federico II, Naples, Italy; ^2^Neuroscience Institute Cavalieri Ottolenghi, Turin, Italy; ^3^Department of Neuroscience “Rita Levi Montalcini”, University of Turin, Turin, Italy; ^4^Brain Science Institute, Korea Institute of Science and Technology, Seoul, Republic of Korea

**Keywords:** cerebellum, fear, anxiety, Duchenne, lead, NDD

The cerebellum has been the focus of attention for many decades regarding its essential role in motor control. Indeed, cerebellar circuitry dysfunction leads to a range of symptoms such as limb movement difficulties, alteration of posture and gait, and difficulties in eye movement and speech control (Stoodley and Schmahmann, 2010). Besides this well-known role, the cerebellum is involved also in cognition and emotion (Leiner et al., 1986; Baillieux et al., 2008). Such diversified level of control by the cerebellum is in part due to functional differences between cerebellar lobules which are anatomically connected to several regions of the brain (Stoodley and Schmahmann, 2010). Given the cerebellar multifunctionality, it is not surprising to find an association between damage or alteration in cerebellar circuits and non-cerebellar diseases (Schmahmann et al., 2007; Stoodley, 2012). Therefore, while exploring such association further, it would be crucial to shift our focus toward investigating the molecular and cellular mechanisms underlying non-cerebellar diseases in the specific cerebellar lobules.

The hypothesis paper from Chin and Augustine provides an overview of research findings in both humans and animal models that suggest the cerebellum as an important player in anxiety disorders. They focused the attention on recent progress in identifying cerebellar topographical locus, circuits, and neuro-modulatory systems in cerebellum-related anxiety behavior. By surveying literatures that connect between the cerebellum and anxiety, the authors also uncover some gaps between them and emphasize the importance of further studies, including how the cerebellar circuit's signal are modified during anxiety and how this information is transmitted to anxiety-related regions.

Hwang et al. review the literatures on the cellular and synaptic mechanisms responsible for the modulation of cerebellar circuits during fear learning and memory. They suggest the contributions of several forms of synaptic plasticity in the cerebellum to fear learning. They also discuss the cerebellar topographical locus and circuits involved in the regulation of fear learning and memory. Specifically, they point out the medial cerebellum, which includes vermal lobules V-VI, VIII, and fastigial nuclei (FN), as the areas involved in the regulation. These areas presumably work together with amygdala, medial prefrontal cortex, hypothalamus, hippocampus, and periaqueductal gray, which are in the fear-related brain circuit. At the conclusion of the review, they suggest several additional areas of study, such as cerebellar efferent pathways and the precise role of the cerebellum in fear learning and memory.

Ribeiro and Sherrard discuss the contribution of cerebellar dysfunction in neuro-developmental disorders (NDDs). They propose the transcription factor Retinoid-related Orphan Receptor alpha (RORα) as a protein with a major causative role in NDDs through cerebellar developmental defect. Notably, RORα regulates multiple events during cerebellar development, including Purkinje cell development and the refinement of synaptic connections, as well as cerebellar maintenance throughout life. In fact, the authors summarize comprehensively the research findings about the relationship between the deficiency of RORα and NDDs. The hypothesis of RORα as a possible candidate for the NDDs pathophysiology via influencing cerebellar development opens the way to act therapeutically on this risk gene target, upregulating the low expression and improving the NDDs symptoms.

Prenatal exposure to heavy metals can lead to NDDs, such as autism spectrum disorders (ASD). Considering the correlation between the occurrence of ASD and abnormal cerebellar development (Wang et al., 2014), Choi et al. in their original research studied whether the exposure to lead during pregnancy alter cerebellar development in mice and induce ASD-like behaviors in the offspring. The authors found that maternal lead exposure caused sex-dependent alterations in cerebellar glial cells and ASD-like behaviors in the offspring: male offspring exhibited microgliosis and repetitive behaviors, whereas female offspring showed astrogliosis but no abnormal repetitive behaviors. The authors also suggested that the sex-dependent difference of the GABA levels in the cerebellum is one of the mechanisms explaining the ASD-like behaviors. These findings highlight the importance of minimizing lead exposure during pregnancy to prevent abnormal cerebellar development and consequent NDDs in offspring.

Duchenne Muscular Dystrophy (DMD) is caused by the mutation in the DMD gene encoding for the dystrophin protein which stabilizes the cellular membrane of muscle cells. Besides the progressive muscle degeneration and weakness which are the main symptoms, DMD patients display also cognitive deficits. Cerebellar Purkinje cells have the highest levels of dystrophin expression in mice, suggesting that cerebellar circuit could be significantly impaired by loss of dystrophin, possibly explaining, at least in part, the motor and cognitive deficits associated with DMD. Kreko-Pierce and Pugh with their study in a *mdx* mouse model of DMD have added precious information regarding alteration in cerebellar signaling in DMD. Using a combination of immunolabelling and whole-cell patch clamp electrophysiology, the authors found a reduced inhibitory effect of PCs to excitatory neurons in the cerebellar nuclei (CbN) in *mdx* mice, particularly during high-frequency activity. Moreover, they reported changes in the excitability of CbN neurons in *mdx* mice with an increased spontaneous firing and a reduced evoked firing. The overall effect of these alterations is a less sensitivity of CbN neurons to both excitatory and inhibitory influences, suggesting an impairment of the output signals from the cerebellar circuits. The altered signals from the cerebellum during development might be the reason of the high association between DMD and NDDs.

This Research Topic gathered many studies that highlight the multiple roles of the cerebellum in addition to the motor control. These studies emphasize the necessity of further studies to understand how the changes of specific microcircuits of the cerebellum can lead to a variety of different diseases.

## Author contributions

PL and EH designed the first draft of the manuscript and revised the manuscript. PL, FM, KT-Y, and EH reviewed and finalized the manuscript. All authors have read and approved the final version of the article for publication.
